# Low-Load Resistance Exercise with Blood Flow Restriction Increases Hypoxia-Induced Angiogenic Genes Expression

**DOI:** 10.2478/hukin-2022-0101

**Published:** 2022-11-08

**Authors:** Rodrigo Volga Fernandes, Valmor Tricoli, Antonio Garcia Soares, Elen Haruka Miyabara, Marcelo Saldanha Aoki, Gilberto Laurentino

**Affiliations:** 1 School of Arts, Sciences, and Humanities, University of São Paulo, SP, Brazil; 2 School of Physical Education and Sport, University of São Paulo, SP, Brazil; 3 Department of Anatomy, Institute of Biomedical Sciences, University of São Paulo, SP, Brazil

**Keywords:** strength training, partial vascular occlusion, skeletal muscle capillarization, vasodilation biomarkers

## Abstract

The aim of the study was to determine whether low-load exercise (LL) with blood flow restriction (LL-BFR) would induce similar changes in expression of genes involved in hypoxia and angiogenesis compared to LL and high-load exercise (HL). Twenty-four males (age: 21.3 ± 1.9 years, body height: 1.74 ± 0.8 m, body mass: 73 ± 1.8 kg) were allocated into three groups: low-load exercise (LL), low-load exercise with blood-flow restriction (LL-BFR), and high-load exercise (HL). For the LL-BFR group a pneumatic cuff was inflated at 80% of the arterial occlusion pressure. All participants performed bilateral knee extension exercise, twice a week, for 8 weeks. LL and LL-BFR groups performed 3-4 sets of 15 reps at 20% 1RM, whilst the HL group performed 3-4 sets of 8-10 reps at 80% 1RM with a 60-s rest interval between sets. The hypoxia-inducible factor-1 alpha (HIF-1α) and beta (HIF-1β), vascular endothelial growth factor (VEGF), neuronal (nNOS), and inducible nitric oxide synthase (iNOS) genes expression were assessed before and after training. HIF-1α and HIF-1β mRNA levels significantly increased in the LL-BFR group and exceeded those elicited by HL and LL groups (p < .0001). VEGF gene expression was increased in both LL-BFR and HL groups, however, LL-BFR elicited a greater increase than LL (p < .0001). nNOS and iNOS genes expression significantly increased in all groups with greatest increases being observed in the LL-BFR group (p < .0001). The findings suggest that LL-BFR induces greater increases in genes expression related to hypoxia and angiogenesis than traditional resistance training.

## Introduction

Angiogenesis is characterized by the formation of new blood vessels from pre-existing capillaries (Egginton, 2011), however, increased skeletal muscle capillarization (e.g., capillary density [CD] and a capillary-to-fiber ratio [C/F]) in response to exercise is a complex process and it is still not fully understood. In addition, hypoxia-inducible factor-1 alpha (HIF-1α) acts as a master regulator for the expression of genes involved in the hypoxia response of most mammalian cells. HIF-1α initiates transcription of various hypoxia-adaptative genes, such as the vascular endothelial growth factor (VEGF) which regulates the process of angiogenesis after the heterodimer formation with HIF-1β (Carmeliet, 2003; Ohno et al., 2012). The VEGF is a potent endothelial specific mitogenic which recruits endothelial cells into hypoxic foci and the avascular area and stimulates their proliferation (Wang and Semenza, 1995).

Evidence suggests that exercise-induced shear stress in the wall of arteries and local hypoxia seem to play an important role in the modulation of genes involved in regulation of capillarization (Hudlick and Brown, 2009; Olfert et al., 2016) and the production of nitric oxide (NO) via the activity of endothelial nitric oxide synthase [eNOS] (Baum et al., 2004; Parise et al., 2020; Patel et al., 2001; Rodriguez-Miguelez et al., 2015). Studies with high-load resistance exercise (HL) and low-load resistance exercise (LL) [e.g., 30 – 50% 1RM] training have demonstrated that changes in muscle mass and strength are accompanied by increases in expression of genes related to hypoxia and angiogenesis, and improvements in skeletal muscle endurance (Holloway et al., 2018a, 2018b; Snijders et al., 2017a, 2017b; Verdijk et al., 2016). For instance, Holloway et al. (2018a) observed increases in HIF-1α, VEGFR2, eNOS gene expression accompanied by increases in skeletal muscle capillarization (CD and C/F) following 12 weeks of HL training (70-80% 1RM). Another study carried out by the same research group showed that microvascular adaptations to resistance training (RT), combined with an increased muscle fibers cross-sectional area (CSA) with sets performed to volitional failure, for 12 weeks, occurred independent of training loads [LL - 30-50% 1RM and HL - 75-90% 1RM] (Holloway et al., 2018b). Thus, performing LL training until muscular failure seems to be an important stimulus to induce microvascular expansion (Holloway et al., 2018b) and skeletal muscle hypertrophy (Mitchell et al., 2012).

Besides traditional resistance training, some acute studies have investigated the effects of low-load resistance exercise with blood flow restriction (LL-BFR) on the expression of genes related to hypoxia and angiogenesis (Ferguson et al., 2018; Larkin et al., 2012). Larkin et al. (2012) observed significant increases in the expression of HIF-1α and VEGF accompanied by increases in two NOS isoforms (i.e., nNOS and iNOS), 4 h and 24 h after LL-BFR. Likewise, Ferguson et al. (2018) observed greater increases in the expression of HIF-1α, VEGF and eNOS 4 h after LL-BFR compared to a LL protocol. These acute studies suggest that increased expression of genes related to hypoxia and angiogenesis may enhance capillary growth in response to LL-BFR.

More recently, other studies reported direct evidence of microvascular adaptations after LL-BFR training (Nielsen et al., 2020; Pignanelli et al., 2020). Pignanelli et al. (2020) showed that LL-BFR and LL training at 30% 1RM, with repetitions until failure induced similar increases in capillary content and capillary contacts in type I muscle fibers, following six weeks of training, revealing no additional effect of blood flow restriction on skeletal muscle capillary angiogenesis when both protocols were performed to muscular failure. Likewise, Nielsen et al. (2020) showed that short-term high-frequency LL-BFR (~23 sessions, one to two daily sessions) at 20% 1RM performed to concentric failure increased skeletal muscle capillary per myofiber and the capillary area following three weeks of training; however, only minor changes were observed in the LL group.

Altogether, the previous studies showed that acute and chronic LL-BFR and HL training increased the expression of genes related to hypoxia and angiogenesis. In addition, LL-induced increases in skeletal muscle capillarization occurred only when exercise was performed until muscular failure. Thus, when LL training is performed with sets of exercise without muscular failure or BFR association, it may potentially attenuate training-induced skeletal muscle adaptations and angiogenesis.

Therefore, the first purpose of this study was to investigate whether the responses in the expression of hypoxia and angiogenesis target genes to LL training would be attenuated when training volume was matched (without failure) to LL-BFR. The second purpose of this study was to investigate whether LL-BFR and HL training programs would result in comparable increases in the expression of hypoxia and angiogenesis target genes in skeletal muscle after 8-wk training. We hypothesized that LL-BFR would contribute to a higher expression of the target genes compared to volume-matched LL. In addition, LL-BFR and HL training would induce similar increases in the expression of these genes and both training protocols would yield more pronounced outcomes than LL training, given that LL was not performed until muscular failure.

## Methods

### Participants and study design

Twenty-four physically active male college students (age 21.3 ± 5.0 years, body height 174.5 ± 6.9 cm, body mass 73.3 ± 12.9 kg) participated in this study. This study was part of a larger project that aimed to investigate whether resistance training programs previously published by Laurentino et al. (2012) would result in comparable expression of hypoxia and angiogenesis target genes (i.e., HIF-1α and HIF-1β; VEGF, nNOS, and iNOS) in skeletal muscle after a training period. Inclusion criteria required that participants had no experience in resistance training at least six months prior to the study. Exclusion criteria included cardiovascular problems, muscle injuries and smoking. Participants were informed of benefits and potential risks of the investigation and gave their written informed consent prior to being part of the present study. This study was conducted according to the Declaration of Helsinki and the University's Research Ethics Committee approved the experimental protocol (FR-254023).

### Resistance training programs

Participants were randomized into one of three RT programs: low-load resistance exercise (LL, n = 9), low-load resistance exercise with blood flow restriction (LL-BFR, n = 8), and high-load resistance exercise (HL, n = 7). Resistance training programs consisted of bilateral knee extensions using a conventional knee extension machine (SL 1030; Riguetto, Campinas, São Paulo, Brazil). Participants were asked to sit comfortably on the machine with their backs fully supported against the backrest. The range of motion was set at 90^⍛^, and each repetition started with the knees at 90^⍛^ flexion. Then, participants were asked to extend their knees until their shanks were parallel to the floor and return to the starting position. During the first 4 weeks of training, the HL group performed three sets of eight repetitions at 80% 1RM, while LL and LL-BFR groups performed three sets of 15 repetitions at 20% 1RM. Exercise volume was then increased to four sets for all the groups for the remainder of the training period (LL = 15785 ± 1791 kg; LL-BFR = 16398 ± 2791 kg; HL = 34563 ± 6097 kg). A 1-min rest interval was allowed between sets throughout the study. The duration of each repetition was established at 4 s (2 s concentric and 2 s eccentric muscle action). In the present study, all exercise protocols were performed without muscle failure. Participants were instructed to consume a light meal before each training session. The LL-BFR group trained with an air cuff placed at the inguinal fold (175 mm width x 920 mm length) and performed knee extension exercise sets at 80% of arterial occlusion pressure, which was sustained during the whole training session, including the rest intervals. Pressure was released immediately after the end of the last exercise set. No adverse effect from the blood flow restriction protocol was reported by any of the participants. They completed an 8-wk resistance training period, with frequency of twice a week. Further details are reported elsewhere (Laurentino et al., 2012).

### Muscle biopsy sample

A unilateral muscle sample was obtained from the vastus lateralis midpoint of the participant´s dominant leg using the percutaneous biopsy technique with suction. All the biopsies were performed in the morning (between 8:00 and 11:00 a.m.) after an overnight (8 h) fasting period. A standardized breakfast meal (~311 kcal; 63.5% CHO, 21.8% proteins, and 14.7% fat) was offered to all the participants 2 h before the biopsies. Immediately after the procedure, the muscle sample was removed from the needle and frozen in liquid nitrogen, for further storage at - 80^⍛^C. The pretest biopsy was performed a week before the familiarization sessions, whereas the posttest biopsy was performed through an incision adjacent to the pretest site (3 cm above the previous incision) 48 h after the last training session. The mRNA expression levels of HIF-1α, HIF-1β, VEGF, nNOS, and iNOS were quantified using the polymerase chain reaction (PCR). For mRNA gene expression analysis, muscle samples from one participant from the LL group, two from the LL-BFR group, and three from the HL group, were withdrawn due to the amount of the muscle sample.

### Gene expression analysis

#### Reverse transcription

Total cellular RNA was isolated from the muscle sample using the Trizol reagent (Invitrogen, Carlsbad, CA). Total RNA (1μg) was typically used in a reaction containing oligo dT (500 μg**^.^**mL^-1^), deoxyribonucleoside triphosphates (10 mM each), 5 first strand buffer, 0.1 M of dithiothreitol, and 200 U of transcriptase (SuperScript II; Invitrogen). Reverse transcription was performed at 70^⍛^C for 10 min followed for 60 min and 95^⍛^C for 10 min.

#### Primer design

Primer sets were designed using Primer Express version 2.0 software (Applied Biosystems, Foster City, CA). Primer sequences were accessed through the GenBank and were checked for specificity using the Nucleotide-Nucleotide BLAST search (Table 1).

**Table 1:** Target genes and sequence of primers

mRNA (genes)	Forward	Reverse
HIF-1α	GCCCCAGATTCAGGATCAGA	TGGGACTATTAGGCTCAGGTGAAC
HIF-1β	CCGGCAGAGAATTTCAGGAATA	TGTCCTGCAGAAGCTGATGG
VEGF	CTTGCTGCTCTACCTCCACCAT	ATGATTCTGCCCTCCTCCTTCT
iNOS	CCCCTTCAATGGCTGGTACA	GCGCTGGACGTCACAGAA
nNOS	GAAATCATCTTGGCCTGATAGCA	GAAGAGACGAACAGAAATGACATTG

#### Real-time PCR

All samples were analyzed in duplicate, and the fluorescence reaction was quantified with an ABI Prism 7300 sequence detector (Applied Biosystems) on the basis of current methodology (Bustin et al., 2009). The amplification analysis was performed with a sequence detection software (Applied Biosystems). Results were expressed using the comparative cycle threshold (Ct) method described in the manufacturer´s User Bulletin n^⍛^2 (Applied Biosystems). The Ct represents the PCR cycle at which an increase in reporter gene fluorescence above a baseline signal can be detected. For each gene of interest, ΔCt values were calculated for all samples as follows: Ct (gene of interest) - Ct (internal control gene). The ribosomal protein large P0 was used as a housekeeping gene (Bustin et al., 2009).

The calculation of the relative changes in the expression levels of one specific gene was performed by subtracting pre to posttest ΔCt values for each experimental group. The values and ranges given were determined as follows: 2^- ΔΔCt^ with ΔΔCt ± SEM (SEM is the SE of the mean ΔΔCt value; User Bulletin n^⍛^ 2; Applied Biosystems). The final values were reported as a fold difference relative to the expression of the pretest values (calculated as 2^-ΔΔCt^), with the pretest values arbitrary set to 1.

### Statistical analysis

Results are presented as means and SD. Data normality and variance equality were assessed through the Shapiro-Wilk and Levene’s tests. A mixed model was performed for each dependent variable (HIF-1α, HIF-1β, VEGF, iNOS, and nNOS) having group (LL, LL-BFR, and HL) and time (pre and posttest) as fixed factors and participants as a random factor. Whenever a significant F value was obtained, a post hoc test with a Tukey adjustment was performed for multiple comparison purposes. Also, analysis based on effect size (ES – Cohen´s) was performed by calculation of the differences between pre- and post-training for all the variables of interest, with the magnitude of the effect being considered as trivial [0.2 – 0.3]; moderate [0.4 – 0.7]; and large [≥ 0.8] (Nakagawa and Cuthill, 2007). The level of significance was set at *p* < 0.05. All the statistical tests were performed using SAS 9.0 for Windows software (SAS Institute Inc., Cary, NC). An initial analysis revealed no significant differences between groups at baseline values for all the dependent variables (*p* > 0.05).

## Results

### HIF-1α gene expression

**Figure 1:**
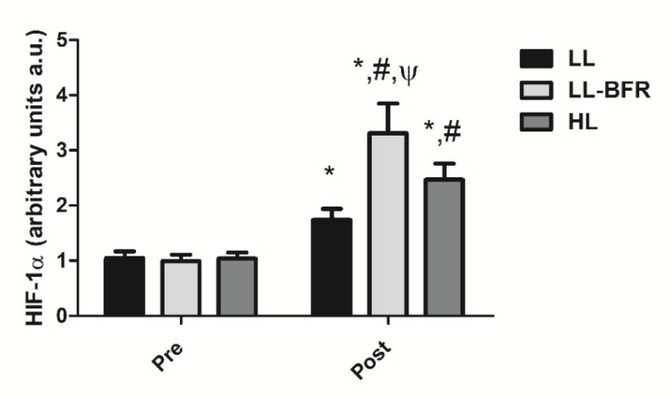
HIF-1α gene expression - arbitrary units [a.u.] (mean ± SD) measured before (Pre) and after the 8-week training period (Post). Low-load resistance exercise (LL), low-load resistance exercise with blood flow restriction (LL-BFR), and high-load resistance exercise (HL). * indicates p < 0.05 for within-groups pre- to post-training comparisons. ^#^ Different from LL at post-training (p < 0.05). ^Ψ^ Different from LL and HL groups at post-training (p < 0.05).

Figure 1 depicts the effects of the RT programs on HIF-1α gene expression before and after the training intervention. There was a condition x time interaction (*p* < .0001), with large effect size. HIF-1α gene expression was significantly increased from pre- to post-training in all groups: LL - pre: 1.05 ± 0.12 to post: 1.74 ± 0.20, CI: 0.30 - 1.08, ES: 4.2, (*p* < .0001); LL-BFR - pre: 0.99 ± 0.12 to post: 3.31 ± 0.54, CI: 1.90 - 2.73, ES: 5.9, (*p* < .0001); and HL - pre: 1.04 ± 0 0.11 to post: 2.47 ± 0.29, CI: 0.98 - 1.86, ES: 6.4, (*p* < .0001). HIF-1α gene expression in the LL-BFR group was significantly higher compared to LL and HL groups at post-training (*p* < .0001). In addition, the increased gene expression in the HL group was significantly higher compared to the LL group at post-training (*p* < .0001).

### HIF-1β gene expression

**Figure 2:**
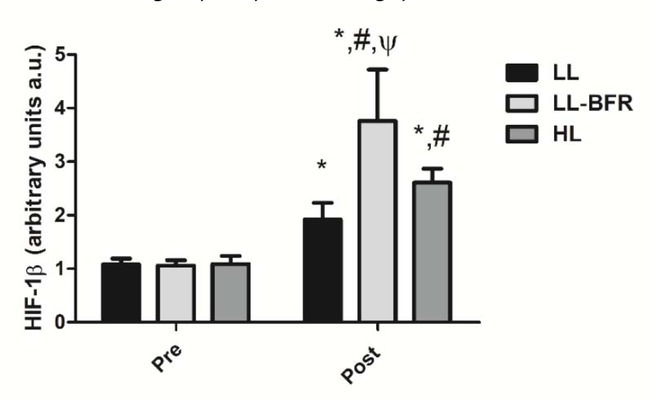
HIF-1β gene expression - arbitrary units [a.u.] (mean ± SD) measured before (Pre) and after the 8-week training period (Post). Low-load resistance exercise (LL), low-load resistance exercise with blood flow restriction (LL-BFR), and high-load resistance exercise (HL). * indicates p < 0.05 for within-groups pre- to post-training comparisons. ^#^ Different from LL at post-training (p < 0.05). ^Ψ^ Different from LL and HL groups at post-training (p < 0.05).

Figure 2 depicts the effects of the RT programs on HIF-1β gene expression before and after the training intervention. There was a condition x time interaction (*p* < .0001) with large effect size. HIF-1β gene expression was significantly increased from pre- to post-training in all groups: LL - pre: 1.08 ± 0.11 to post: 1.92 ± 0.31, CI: 0.22 - 1.48, ES: 3.6, (*p* < .0001); LL-BFR - pre: 1.06 ± 0.10 to post: 3.76 ± 0.96, CI: 2.02 - 3.36, ES: 3.9, (*p* < .0001); and HL - pre: 1.09 ± 0.15 to post: 2.61 ± 0.26, CI: 0.79 - 2.22, ES: 7.1, (*p* < .0001). HIF-1β gene expression in the LL-BFR group was significantly higher compared to LL and HL groups at post-training (*p* < .0001). In addition, the increased gene expression in the HL group was significantly higher compared to the LL group at post-training (*p* = 0.043).

### VEGF gene expression

**Figure 3:**
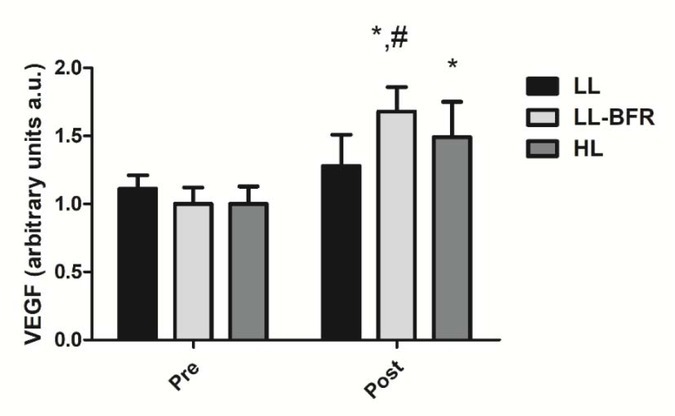
VEGF gene expression - arbitrary units [a.u.] (mean ± SD) measured before (Pre) and after the 8-week training period (Post). Low-load resistance exercise (LL), low-load resistance exercise with blood flow restriction (LL-BFR), and high-load resistance exercise (HL). * indicates p < 0.05 for within-groups pre- to post-training comparisons. ^#^ Different from LL group at post-training (p < 0.05).

Figure 3 depicts the effects of the RT programs on the VEGF gene expression before and after the training intervention. There was a condition x time interaction (*p* < .0001) with large effect size. The VEGF gene expression was significantly increased from pre- to post-training in the LL-BFR and HL groups: LL - pre: 1.11 ± 0.10 to post: 1.28 ± 0.23; CI: 0.08 – 0.4035, ES: 0.9 (*p* = 0.353), LL-BFR - pre: 1.00 ± 0.12 to post: 1.68 ± 0.18, CI: 0.42 - 0.93; ES: 4.4, (*p* < .0001); and HL - pre: 1.00 ± 0.13 to post: 1.49 ± 0.26; CI: 0.21 - 0.76, ES: 2.3, (*p* < .0001). The increase in the VEGF gene expression in the LL-BFR group was significantly higher compared to the LL group at post-training (*p* = 0.001).

### nNOS gene expression

**Figure 4:**
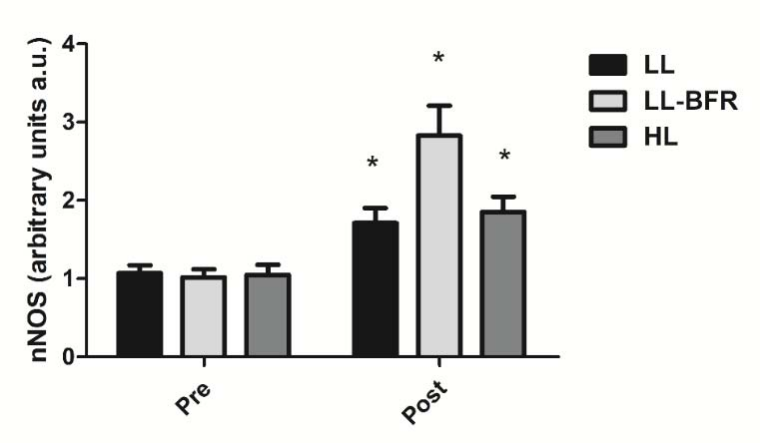
nNOS gene expression - arbitrary units [a.u.] (mean ± SD) measured before (Pre) and after the 8-week training period (Post). Low-load resistance exercise (LL), low-load resistance exercise with blood flow restriction (LL-BFR), and high-load resistance exercise (HL). * indicates p < 0.05 for within-groups pre- to post-training comparisons.

Figure 4 depicts the effects of the RT programs on nNOS gene expression before and after the training intervention. There was a condition x time interaction (*p* < .0001) with large effect size. nNOS gene expression was significantly increased from pre- to post-training in all groups: LL - pre: 1.07 ± 0.10 to post: 1.70 ± 0.19; CI: 0.34 - 0.94, ES: 4.3, (*p* < .0001); LL-BFR - pre: 1.02 ± 0.10 to post: 2.83 ± 0.38; CI: 1.49 - 2.13, ES: 6.5, (*p* < .0001); and HL - pre: 1.05 ± 0.13 to post: 1.85 ± 0.20; CI: 0.45 - 1.13, ES: 4.7, (*p* < 0.0001). nNOS gene expression in the LL-BFR group was significantly higher compared to LL and HL groups at post-training (*p* < .0001).

### iNOS gene expression

**Figure 5:**
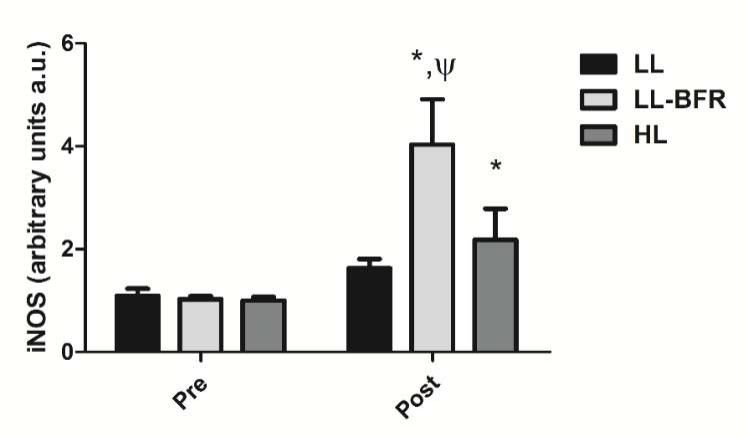
iNOS gene expression - arbitrary units [a.u.] (mean ± SD) measured before (Pre) and after the 8-week training period (Post). Low-load resistance exercise (LL), low-load resistance exercise with blood flow restriction (LL-BFR), and high-load resistance exercise (HL). * indicates p < 0.05 for within-groups pre- to post-training comparisons. ^Ψ^ Different from LL and HL groups at post-training (p < 0.05).

Figure 5 depicts the effects of the RT programs on iNOS gene expression before and after the training intervention. There was a condition x time interaction (*p* < .0001) with large effect size. iNOS gene expression was significantly increased from pre- to post-training in the LL-BFR and HL groups: LL – pre: 1.09 ± 0.18 to post: 1.63 ± 0.18; CI: 0.09 – 1.16, ES: 3.2 (*p* = 0.135); LL-BFR - pre: 1.03 ± 0.06 to post: 4.03 ± 0.88; CI: 2.33 -3.66; ES: 4.8, (*p* < .0001); and HL - pre: 1.00 ± 0.07 to post: 2.18 ± 0.61; CI: 0.46 - 1.89, ES: 2.7, (*p* < .0001). iNOS gene expression in the LL-BFR group was significantly higher compared to LL and HL groups at post-training (*p* < .0001).

## Discussion

The present study investigated whether LL-BFR training would yield a higher increase in the expression of genes related to hypoxia and angiogenesis compared to volume-matched LL training. In addition, we tested whether LL-BFR and HL training programs would result in a similar expression profile of these genes. The data indicated that LL-BFR training yielded a greater up-regulation of genes related to hypoxia and angiogenic signaling in skeletal muscle compared to both LL and HL training after 8 weeks.

The results reported herein showed that the HIF (isoforms 1α and 1β) mRNA level was significantly increased in all training groups; however, the magnitude of increases in HIF-1α and HIF-1β in the LL-BFR group exceeded that elicited by LL and HL groups. HIF-1α initiates transcription of various hypoxia-adaptative genes involved in angiogenesis (i.e., VEGF, nNOS, iNOS, and eNOS), after formation of the active heterodimer with HIF-1β (Ohno et al., 2012). The greater changes in HIF-1α, HIF-1β and VEGF genes expression in the LL-BFR group compared to the volume-matched LL group may be due to the further reduction in oxygen saturation and oxygen tension, given that the restriction pressure applied in the LL-BFR group was kept throughout the exercise session, including rest intervals across sets (Larkin et al., 2012). For example, Larkin et al. (2012) showed that deoxygenated hemoglobin (HHb) and total hemoglobin (THb) in vastus lateralis during LL-BFR was significantly greater compared to the LL condition. These changes were accompanied by increased VEGF gene expression at 4 h post-exercise in response to LL-BFR.

Additionally, in this study, the magnitude of increases in HIF-1α and HIF-1β genes expression from the LL-BFR group was greater when compared to the HL group (234% and 254% vs. 136% and 139%). Congruent with our findings, Tanimoto et al. (2005) demonstrated a lower muscle oxygenation level during LL-BFR at 30% 1RM than both resistance exercise protocols performed at 50% 1RM and 80% 1RM. In addition, one recent study conducted by Ilett et al. (2019) reported that tissue muscle oxygenation (TMO) during exercise was significantly reduced across sets for HL and LL-BFR, with no significant change in LL. However, during rest intervals between sets, TMO was recovered to the baseline level only for the HL group, while it remained unchanged during the entire LL-BFR protocol. These findings support the contention that a HL protocol may cause smaller decreases in TMO during exercise and rest intervals due to relative local hyperoxygenation.

To date, only two acute studies have investigated the effects of LL-BFR on hypoxia-related gene expression, using a randomized crossover design (Ferguson et al., 2018; Larkin et al., 2012). In both studies, HIF-1α and VEGF gene expression were increased in the LL-BFR group 2 h and 4 h post-exercise, and were significantlygreater than the LL protocol. Furthermore, Ferguson et al. (2018) reported an acute increase of 4.6-fold in VEGFR2 gene expression in the LL-BFR when compared to the LL condition. The current findings corroborate previous acute studies, providing clear evidence of the role of hypoxia in up-regulation of genes related to angiogenesis and capillarization after 8 weeks of training.

The potential effect of LL-BFR on microvascular adaptations has been shown in few studies (Hunt et al., 2013; Pignanelli et al., 2020). Hunt et al. (2013) observed enhanced capillary filtration after 6-wk LL-BFR training at 40% 1RM, with no significant change following the LL protocol. Recently, another study with LL-BFR training using skeletal muscle biopsy reported direct evidence of microvascular adaptations (Pignanelli et al., 2020). Pignanelli et al. (2020) showed that LL-BFR and LL training at 30% 1RM, with repetitions until failure, induced similar increases in capillary content of type I muscle fibers and capillary contacts, following 6 weeks of training. However, it is important to underscore that the similarity of increases in microvascular adaptations in the LL and LL-BFR groups may have been due to the LL protocol to be performed at 30% 1RM and with repetitions until failure. Supporting the contention of repetitions until failure associated to the LL protocol on microvascular adaptations, another study compared effects of LL until muscular failure (30-50% 1RM) versus HL (75-90% 1RM) on genes expression related to hypoxia and angiogenesis and skeletal muscle endurance (Holloway et al., 2018b). After the training intervention, HIF-1α, VEGFR2, and eNOS expression and capillarization (CD and C/F) were increased with concomitant increases in skeletal muscle fiber CSA in both groups. This study showed that the expression of hypoxia-related genes and microvascular adaptations in the LL and HL groups occurred independently of training loads. Herein, we showed that HIF-1α, HIF-1α, and VEGF genes expression in LL-BFR and HL groups were greater than in the LL group. It is plausible to suggest that, in our study, the load (20% 1RM) and non-failure repetitions in the LL group would explain different responses in the expression of genes related to hypoxia compared to LL-BFR and HL groups.

The shear stress, hypoxia, and muscle contraction have been implicated in increased nitric oxide (NO) in response to exercise, and these stimuli may also trigger factors related to angiogenesis (Casey et al., 2017; Melikian et al., 2009). We showed that the expression of nNOS and iNOS isoforms was increased following the training intervention (nNOS = LL: 60%, LL-BFR: 178%, and HL: 75%; and iNOS = LL-BFR: 291% and HL: 117%) with no significant change in iNOS gene expression in the LL group. This is the first study that investigated gene expression of different NOS isoforms in response to LL, LL-BFR, and HL training and found LL-BFR-induced greater increases in nNOS (~2.9- and 2.3-fold) and iNOS gene expression (~6- and 2.4-fold), compared to those from LL and HL groups. Congruent with our data, Larkin et al. (2012) reported increased nNOS expression 4 h and iNOS expression 24 h postexercise in the LL-BFR compared to the LL protocol. Those authors associated the increases in NOS expression with significant changes in the VEGF expression. Likewise, Ferguson et al. (2018) observed increased eNOS and VEGF expression 4 h postexercise following the LL-BFR protocol. Additionally, we demonstrated that the increase in nNOS and iNOS mRNA levels resulting from LL-BFR were 2.3-fold and 2.4-fold higher than in the HL group. Although Larkin et al. (2012) and Ferguson et al. (2018) investigated acute responses to LL-BFR, it was suggested that the results of the present study could be associated with larger HIF-1α and HIF-1β expression observed in the LL-BFR group after an 8-week training period.

The current study has some limitations that should be considered: 1) our findings are not supported at the protein level, and that needs to be taken into consideration, and 2) we did not measure direct angiogenesis biomarkers (i.e., number of capillary/fiber ratio, capillary contacts, and individual capillary-to-fiber ratio [C/F]). Despite this fact, we demonstrated that gene expression of angiogenesis biomarkers and hypoxia modulators were augmented in response to LL-BFR and HL training, with the occlusive stimulus overtaking those from LL.

## Conclusions

We demonstrated herein that gene expression of microvascular and vasodilation biomarkers (VEGF and iNOS) in the LL group was unchanged following a resistance training intervention. Divergent from previous research, it is possible that total exercise volume exhibited by the LL group (i.e., 3-4 sets of 15 reps, at 20% 1RM) and a non-failure exercise protocol were insufficient to stimulate significant increases in microvascular and vasoactive enzyme biomarkers gene expression. Furthermore, the findings suggest that LL-BFR induces greater increases in genes expression related to hypoxia and angiogenesis than traditional resistance training.
